# Molecular Examination of *Trichomonas vaginalis* Infection and Risk of Prostate Cancer in the Biopsy of Patients with Different Prostate Lesions

**DOI:** 10.4314/ejhs.v31i2.5

**Published:** 2021-03

**Authors:** Zeinab Kamarkhani, Raheleh Rafiei-Sefiddashti, Leila Haghighi, Alireza Badirzadeh, Ramtin Hadighi

**Affiliations:** 1 Department of Parasitology and Mycology, School of Medicine, Iran University of Medical Sciences, Tehran, Iran

**Keywords:** Trichomonas vaginalis, Prostatic Hyperplasia, Prostate cancer, PCR

## Abstract

**Background:**

Trichomoniasis is a sexually transmitted infectious disease caused by a flagellated protozoa, Trichomonas vaginalis (T.vaginalis) and is often asymptomatic in men. Benign prostatic hyperplasia (BPH) and prostate cancer (PCA) are the most common urological diseases in the elderly. Scientists have proposed various factors which trigger prostate cancer, including sexually transmitted diseases. Thus, this study aimed to evaluate the potential role of T. vaginalis as a risk factor for various prostate lesions such as hyperplasia and prostate cancer.

**Methods:**

A total of 250 paraffin-embedded of different prostate lesion biopsies were analyzed by Polymerase Chain Reaction (PCR) using the beta-tubulin gene for identifying T. vaginalis.

**Result:**

All 250 pathologic specimens were negative for this parasite by using PCR technique.

**Conclusion:**

It seems that T. vaginalis may have not had a causative role for different prostate lesions and it seems proposed PCR technique is an insufficient method to find the parasite in paraffin-embedded tissues. Therefore, other diagnostic techniques to identify the parasite in biopsy samples are suggested.

## Introduction

*Trichomonasvaginalis* (*T. vaginalis*) is a flagellate protozoan that causes trichomoniasis which is one of the most common sexually transmitted non-viral infections(1,2). Trichomoniasis is usually asymptomatic in men but can cause urethritis and prostatitis and may produce symptoms such as shown that *T. vaginalis* causes 180 million new cases per year worldwide. In Iran, the prevalence of this parasite has been reported between 2 to 8% and likely up to30% in high-risk populations(4). Detection based on culture tests has shown little sensitivity in return PCR method with high sensitivity (1) and diagnosis is typically made by the wet mount microscopic method because it is fast, effective, and very simple though culture has been used as a gold standard to diagnose this protozoan.

Benign prostatic hyperplasia (BPH) and prostate cancer (PCA) are the most common urological diseases in the elderly(5). Prostate cancer is the second leading cause of cancer mortality among men with the highest prevalence in the United States and China(6,7). The prevalence of this cancer has increased from 2007 to 2017 years to 2876 (9/90%) and has a direct density-ratio to age in Iran (8,9). In other words, it can be said mortality and morbidity were varied by geographic regions and age(10).

There are various factors which cause prostate cancer and sexually transmitted disease; trichomoniasis might be one of them (11). Iqbal et al. examined the presence of *T. vaginalis* in new prostate tissue samples with the beta-tubulin gene, which was able to detect the presence of protozoa. QasimShahran et al. used the samples of prostate paraffin biopsy with PCR and beta-tubulin gene (195 bp length) and failed to detect parasite may be because of damage to DNA samples in deparaffinization procedure, so a smaller length gene was chosen in this study(12).

The purpose of this study was identifying *T. vaginalis* in various prostatic paraffin biopsies including hyperplasia and prostate cancer with PCR by using the beta-tubulin gene.

## Material and Methods

The Paraffin-embedded Prostate Biopsy blocks (PPBs) of 250 patients were gathered from the Pathology Department of Baqiyatollah al-Azam Hospital in Tehran, Iran(ethic code No: IR.IUMS.FMD.REC.1397.064). Total of 250 PPBs were collected from patients with prostate lesions based on the pathologist's diagnosis, including 100 benign prostatic hyperplasia, 100 prostate cancer, and 50 healthy lesions without specific pathology lesions. Then, all samples were coded, anonymized and examined for the presence of *T.vaginalis* genes with PCR technique. Briefly, the PPBs were deparaffinized using xylol and DNA extracted using the Gene ALL DNA Extraction Tissue Kit (PishgamBiotech Company), according to the manufacturer's protocol. Then, the samples were examined by using PCR to find *T. vaginalis* beta-tubulin (BTUB) gene. PCR-specific primers wereBTUB9, 5′-CAT TGA TAA CGA AGC TCT TTA CGA-3′ and BTUB2, 5′-GCA TGT TGT GCC GGA CAT AAC CAT-3′ which were used in different studies(1, 5). *T. vaginalis* (donated from Pasteur Institute of Iran) and distilled water were used as positive and negative controls in each run, respectively. Finally, the products were run along with a DNA ladder (100 bp, Jena Bioscience, Jena, Germany) on 2.5% gel agarose containing ethidium bromide, and visualized under UV light. PCR products of 112 bp fragment length were positive for *T. vaginalis*.

## Results

**Figure 1:**
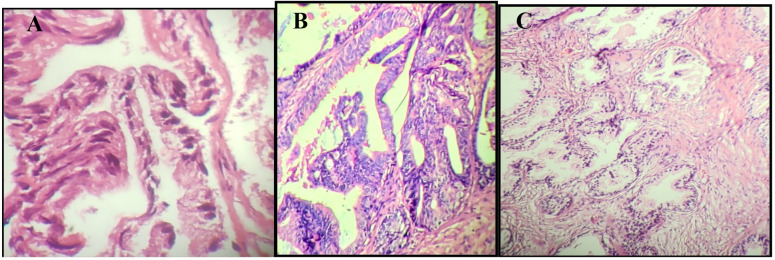
Pathology slides of (A) benign prostatic hyperplasia, (B) prostate cancer and (C) healthy prostate tissue without specific pathology lesions. All slides stained by the hematoxylin-eosin method.

**Figure 2:**
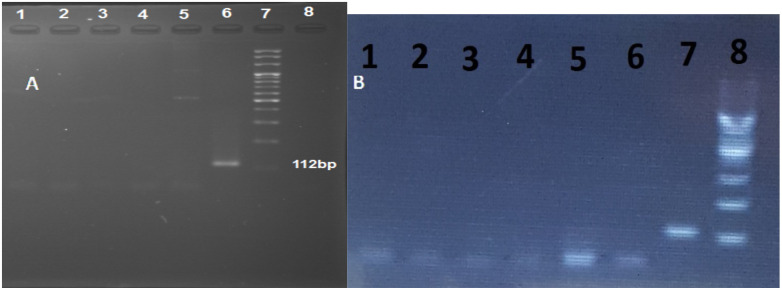
Polymerase chain reaction (PCR) results of BTUB9 and BTUB2 of patients with (A) benign prostatic hyperplasia and (B) prostate cancer on 2.5% agarose gel. Columns 1 to 5 are patients, column 6 positive control (T. vaginalis), column 8 negative control (distilled water), and column 7, 100 bp DNA ladder. All columns were negative for the presence of T. vaginalis.

Pathological slides, including 100 prostate cancer samples, 100 benign prostatic hyperplasia samples, and 50 healthy lesions without specific pathology lesions, were selected for the study (Figure 1). All 250 specimens, including no-pathologic lesions, benign prostatic hyperplasia, and prostate cancer, were negative for *T. vaginalis* DNA (Figure 2). Statistical analysis has shown that the mean age for patients was 67 years old.

## Discussion

Nowadays, the prevalence of prostate cancer is growing among men, and previous studies have suggested that one of the possible causes of prostate inflammation may be *T. vaginalis*, a protozoan parasite (11). This incidence is lower in Asian countries than in western countries, which may be due to nutrition, genetics, lifestyle or environmental factors (13). Prostate cancer is the third most common visceral cancer in Iranian men. The prevalence of this cancer is higher than in South-Central Asian and East Asian countries lower than in North America, South Europe and Eastern Europe and has increased since the late 1980s. The cause of this increase is not entirely clear. However, it appears to have been influenced by new diagnostic methods and PSA test to screen for prostate cancer(14). Various factors can cause prostate lesions, like sexually transmitted diseases, including *Chlamydia trachomatis*, *Neisseria gonorrhoeae* and *T. vaginalis* (5).

A study was performed on 33 patients with symptoms of urinary tract infection by Lee et al. in South Korea and identified 7 cases of *T. vaginalis* by PCR method on urine samples. It was demonstrated that PCR can detect *T. vaginalis*DNA even if there is only one *T. vaginalis* cell in samples(15). Iqbal et al. evaluated the presence of *T. vaginalis* in fresh specimens of prostate tissue and the level of antibody against it in patients with benign prostatic hyperplasia lesions in 171 patients and it was found 42 positive results by PCR technique and 37 positive results by immunocytochemistry. Thus, there was a significant relationship between protozoa and benign prostatic hyperplasia (5). Although community type is so important in the number of positive results but also fresh specimens of prostate tissue may be another factor. In other words, it can be said that fresh specimens may be better samples for finding this protozoan. Yow et al. detected DNA of *Mycoplasma genitalium, Ureaplasmaurealyticum, Herpes simplex* virus in paraffin-embedded prostate biopsy but the DNA of *T. vaginalis, Ureaplasma parvum, Chlamydia trachomatis* and human papillomavirus was not funded (16)which may confirm the difficultness and impossibility of finding parasite DNA with PCR technique in paraffin-embedded samples.

*T. vaginalis* infection and the risk of developing prostate cancer was studied by Shui et alin 146 men with advanced prostate cancer and 181 controls as a case-control study. Measurement of serum antibody level indicated the antibody level against *T. vaginalis* were not associated with increased risk of metastatic or lethal prostate cancer (17). In a prospective study by Fowk et al. in 296 African-American men, there was no significant association between *T. vaginalis* and the risk of prostate cancer by ELISA tests on sera(18). In another review and meta-analysis article, 5590 men with Trichomoniasis was entered and according to the combined estimation, no significant relationship was found between *T. vaginalis* and prostate cancer prevalence (19). In this study, we were unable to find the DNA of the parasite in all prostate lesions. Thus, it can be concluded that this sexually transmitted disease has little effect on increasing the incidence of prostate lesions. Moreover, PCR may not be a good test for diagnosis of this parasite in paraffin-embedded samples. As a result, further researches are needed to confirm the role of *T. vaginalis* in prostate inflammation and prostate cancer. We have suggested a serological test for finding out the relationship between *T. vaginalis* and prostate cancers.

It seems that *T. vaginalis* may have not been a causative agent for different prostate lesions. We suggested that PCR is not a good technique to find the parasite in paraffin-embedded tissues. Therefore, other diagnostic techniques are suggested to identify the parasite in these biopsy samples.
